# Identification of Putative Ortholog Gene Blocks Involved in Gestant and Lactating Mammary Gland Development: A Rodent Cross-Species Microarray Transcriptomics Approach

**DOI:** 10.1155/2013/624681

**Published:** 2013-10-30

**Authors:** Maricela Rodríguez-Cruz, Ramón M. Coral-Vázquez, Gabriel Hernández-Stengele, Raúl Sánchez, Emmanuel Salazar, Fausto Sanchez-Muñoz, Sergio Encarnación-Guevara, Jorge Ramírez-Salcedo

**Affiliations:** ^1^Unit of Medical Research in Nutrition, Hospital de Pediatría, Centro Médico Nacional Siglo XXI, Instituto Mexicano del Seguro Social, Avenida Cuauhtémoc 330, Col. Doctores, Delegación Cuauhtémoc, 06725 Mexico City, Mexico; ^2^Sección de Estudios de Posgrado e Investigación, Escuela Superior de Medicina, Instituto Politécnico Nacional, Plan de San Luis y Díaz Mirón s/n, Col. Casco de Santo Tomas, Delegación Miguel Hidalgo, 11340 Mexico City, Mexico; ^3^Subdirección de Enseñanza e Investigación, Centro Médico Nacional 20 de Noviembre, Instituto de Seguridad y Servicios Sociales de los Trabajadores del Estado, San Lorenzo 502 (2 piso), Col. Del Valle, Delegación Benito Juárez, 03100 Mexico City, Mexico; ^4^Genomic Sciences Center, Universidad Nacional Autónoma de México, Avenida Universidad s/n, Col. Chamilpa, 62210 Cuernavaca, Morelos, Mexico; ^5^Department of Immunology, Instituto Nacional de Cardiología Ignacio Chávez, Juan Badiano No. 1 Col. Sección XVI, 140080 Tlalpan, Mexico City, DF, Mexico; ^6^Microarray Unit, Instituto de Fisiología Celular, Universidad Nacional Autónoma de México, Circuito Exterior s/n, Ciudad Universitaria, Delegación Coyoacán, 04510 Mexico City, Mexico

## Abstract

The mammary gland (MG) undergoes functional and metabolic changes during the transition from pregnancy to lactation, possibly by regulation of conserved genes. The objective was to elucidate orthologous genes, chromosome clusters and putative conserved transcriptional modules during MG development. We analyzed expression of 22,000 transcripts using murine microarrays and RNA samples of MG from virgin, pregnant, and lactating rats by cross-species hybridization. We identified 521 transcripts differentially expressed; upregulated in early (78%) and midpregnancy (89%) and early lactation (64%), but downregulated in mid-lactation (61%). Putative orthologous genes were identified. We mapped the altered genes to orthologous chromosomal locations in human and mouse. Eighteen sets of conserved genes associated with key cellular functions were revealed and conserved transcription factor binding site search entailed possible coregulation among all eight block sets of genes. This study demonstrates that the use of heterologous array hybridization for screening of orthologous gene expression from rat revealed sets of conserved genes arranged in chromosomal order implicated in signaling pathways and functional ontology. Results demonstrate the utilization power of comparative genomics and prove the feasibility of using rodent microarrays to identification of putative coexpressed orthologous genes involved in the control of human mammary gland development.

## 1. Introduction

Mammals are the only animals that secrete a complex fluid from an elaborated skin gland to provide both innate protection and nourishment for their newborn. There are more than 4,000 species of mammals with striking similarities in the structure and function of their mammary glands as well as in their unique milk components such as the caseins, *α*-lactalbumin, lactoferrin, lactose, and milk fat. Nevertheless, variations are exhibited in the arrangement and numbers of mammary gland, milk composition, and suckling strategies. Mammary gland development begins at puberty and is maintained throughout pregnancy until lactation. During these last stages, development compromises numerous overlapping programs such as branching morphogenesis, inductive stromal-epithelial interactions, programmed cell death, extracellular matrix remodeling, and hormone action [1].

Current knowledge of the molecular regulation of mammary development and lactation has largely been derived from the dissection of signaling networks in cell culture systems and phenotypic characterization of genetically altered mice as well as genomewide approaches such as microarrays. Nonetheless, to date, regulation of mammary gland development during pregnancy and lactation is incompletely understood. Lactation is regarded as one of the most remarkable products of evolution whose processes include the development of mammary tissue as well as the synthesis and secretion of milk [2]. Consequently, despite the fact that the development of mammary tissue and the synthesis and secretion of milk are considered as complex dynamic physiological processes, both must preserve overall common characteristics among mammals.

Considering the underlying assumption that important biological functions are often conserved across species, genes expressed across multiple species are likely to have conserved functions [3]. Given the completion of the DNA sequence of the human, mouse, and rat genomes [4], genes identified in microarray studies can be readily compared across species with respect to orthologous genes [5]. Therefore, a cross-species hybridization (CSH) experiment could provide significant information concerning probable conserved gene networks among mammals. 

In a CSH experiment, there is hybridization of RNA from one or more (target) species to a microarray that contains DNA (cDNA or oligomers) from another (reference) species and represents a valuable tool for the identification of orthologous genes. Thus, a CSH microarray analysis offers the possibility of furthering our understanding of cross-species commonalities and differences that could lead to more effective use of animal models to understand the regulation of mammary gland development at the molecular level [6]. Dissection of unique patterns of expression of orthologous clusters of genes among species throughout distinct physiological time points along pregnancy and lactation could prove useful in the integrative analysis of the information available for discerning the molecular events underlying the regulation of mammary gland development and function that lead to milk synthesis.

In this study, bioinformatics techniques were applied to transcriptomic data. These data resulted from heterologous microarrays of target RNA samples derived from rat mammary gland during distinct stages of pregnancy and lactation in order to extrapolate and enhance the understanding on transcriptional module networks or coregulated functional gene groups conserved in rodents and in the development of the mammary gland in humans.

## 2. Materials and Methods

### 2.1. Experimental Animals and Tissue Collection

Fifteen female Sprague Dawley rats were obtained from the Animal Care Facility of Centro Médico Nacional Siglo XXI of the Mexican Institute of Social Security (IMSS) in Mexico City. Animals were housed at 22 ± 2°C with a 12 h light/dark cycle with free access to water, and a purified diet was administered *ad libitum* during pregnancy and lactation. Dietary composition was previously reported by our group [7]. At 14 weeks of age, rats were randomly assigned to five groups representing distinct time points in mammary gland development: virgin (V), day 5 (P5) and, day 14 (P14) of pregnancy and day 1 (L1), and day 12 (L12) of lactation. Three rats were included in each group. Rats were mated and the same diet was administered during pregnancy and lactation. The day on which sperm was identified in vaginal smears was designated as day 1 of pregnancy and the day of parturition was designated as day 1 of lactation. Pregnant rats were housed individually. Litters were adjusted to eight pups per dam. No gender differentiation was made. Pups remained with their mother to stimulate milk synthesis. Rats were euthanized, and whole mammary tissue was removed from V, P5, P14, L1, and L12 rats. Tissue was immediately frozen in liquid nitrogen and stored at −70°C for subsequent total RNA isolation or histological analysis.

### 2.2. Microarray Analysis

#### 2.2.1. Total RNA Isolation

Total RNA was isolated from tissue (0.1-0.2 g) using TRIzol (Invitrogen, Carlsbad, CA, USA) following the method of Chomczynski and Sacchi [8]. Total RNA from mammary tissue was isolated from three different animals of each physiological period (V, P5, P14, L1, and L12), pooled, and kept in aliquots for later determination of purity and integrity. Total pooled RNA was used for microarray analysis and quantitative real-time PCR.

Four microarray datasets generated using the custom-designed *Mus musculus* oligonucleotide array containing 65-mer probe sets representing 22,000 transcripts (Microarray Unit, Cellular Physiology Institute, UNAM, Mexico City) were analyzed. Each dataset represented distinct time points in mammary gland development such as P5, P14, L1, and L12. Histologically, the mammary proliferative stage is represented by P5, the secretory differentiation stage by P14, early lactation by L1, and full lactation by L12. Design of the microarray experiments is presented in Table S1 in supplementary materials available online at http://dx.doi.org/10.1155/2013/624681.

#### 2.2.2. Probe Preparation and Hybridization to Arrays

Ten *μ*g of total pooled RNA was reverse transcribed into cDNA incorporating dUTP-Cy3 or dUTP-Cy5 and using the CyScribe First-Strand cDNA labeling kit (Amersham Biosciences, Piscataway, NJ, USA). Using hybridization solution UniHyb (TeleChem International Inc., Sunnyvale, CA, USA), equal quantities of labeled cDNA were hybridized to the M22 K_01 microarray for 14 h at 42°C. Four hybridization assays were carried out as follows: (a) the fluorophore used was dUTP-Cy3 for control nonpregnant virgin rats (V) and dUTP-Cy5 for P5, (b) dUTP-Cy3 for V and dUTP-Cy5 for P14, (c) dUTP-Cy3 for V and dUTP-Cy5 for L1, and (d) dUTP-Cy3 for V and dUTP-Cy5 for L12. Each hybridization assay was carried out in triplicate. 

Data acquisition and analysis of array images were performed in ScanArray 4000 with its accompanying software ScanArray 4000 from Packard BioChips.

### 2.3. Data Analysis

#### 2.3.1. Global Analysis: Overview of Gene Expression

Microarray data analysis was performed with free software genArise, which was developed in the Computing Unit of the Cellular Physiology Institute of the UNAM (http://www.ifc.unam.mx/genarise/). The goal of genArise is to identify which genes show good evidence of being differentially expressed. The software identifies differentially expressed genes by calculating an intensity-dependent *z*-score. Elements with a *z*-score >1.5 standard deviations are considered to be significantly and differentially expressed genes. 

The complete set of raw Excel data files have been deposited at Gene Expression Omnibus (GEO) and are available on the GEO website (ID GEO GSE22545). 

#### 2.3.2. Clustering Analysis for Gene Expression

Gene lists were generated by a set of multiple comparisons among the distinct developmental stages and intersection in Venn diagrams. Two-way hierarchical clustering with average linkage and a range of 5 to 15 K-means classifications were used to group our time series data using open source software Cluster v3.0 [9]. Java TreeView was used to display the clustering results as dendogram or heat map representations. We adopted the procedure as described in [10] to code the mean expression of a cluster at each stage as flat, decreased, and increased and converted it to numerical representation. 

#### 2.3.3. Determination of Orthologous Genes

Putative orthologous genes in rat, mouse, and human were identified from a genome comparative search with Roundup (http://rodeo.med.harvard.edu/tools/roundup). Roundup is an ortholog and phylogenetic profile retrieval tool backed by a massive repository of orthologous and associated evolutionary distances that were built using the reciprocal smallest distance algorithm [11]. The search was done with a stringent blast *E*-value threshold of 1.0 × 10^−5^ and a divergence threshold of 0.2.

#### 2.3.4. Gene Ontology Analysis

The DAVID 2.0 Functional Annotation Tool (http://david.abcc.ncifcrf.gov/summary.jsp) was used to sort and arrange the similar, redundant, and heterogeneous annotation contents from a set of genes into defined functional groups. In the case of insufficient gene ontology information, published data on orthologous genes was used to classify the gene into a functional category.

#### 2.3.5. Pathway Analysis

Pathway mapping was accomplished using the Kyoto Encyclopedia of Genes and Genomes (KEGG) database of biological systems that integrates genomic, chemical, and systemic functional information (http://www.genome.jp/kegg/kegg2.html).

#### 2.3.6. Interaction Network Analysis

Gene lists were converted to Human SwissProt IDs using tables from the Ensembl database, release 42 [12]. For each list of Human SwissProt IDs, interactions between those gene products were obtained from Online Predicted Human Interaction Database (OPHID) and postprocessed using custom scripts to determine all linkages in the network and to generate a network file. This network file was then explored using NAVIGaTOR v2.0.15, a program for large network analysis (http://ophid.utoronto.ca/navigator/index.html).

#### 2.3.7. Transcription-Factor Binding-Site Prediction

Transcription-factor-binding site (TFBS) prediction was accomplished using CORE_TF (Conserved and Over-Represented Transcription Factor binding sites), a web-based tool that identifies overrepresented TFBS in promoters from coexpressed genes aided by the evaluation of cross-species conservation.

#### 2.3.8. Quantitative Real-Time PCR

We utilized qRT-PCR for validation of microarray results. We measured the relative transcript levels of 14 target genes, and five genes commonly used as references such as Glyceraldehyde-3-phosphate dehydrogenase (*Gapdh*), beta-actin (*Actb*), and ribosomal large protein P0 (*Rlp0*) were used as high abundance internal controls as well as splicing factor arginine/serine-rich 1 (*Sfrs1*) and hypoxanthine guanine phosphoribosyl transferase 1 (*Hprt1*) as medium- and low-abundance internal controls, respectively (Table S2).

Quantitative real-time PCR was performed in a 20 *μ*L reaction with 5.0 *μ*L from 1/4 reverse transcription dilution using the LightCycler Probes Master mix (Roche Diagnostics, Mannheim, Germany) containing 0.2 *μ*M of mRNA-specific primers and 0.1 *μ*M corresponding UPL probe into LightCycler microplate wells under reduced light conditions. Each sample was run in triplicate. 

### 2.4. Calculations and Statistics

The results are expressed as mean ± SEM of at least three individual experimental observations. Data were tested for normality of distribution by the Kolmogorov-Smirnov test. Statistically significant differences among experimental groups (between the mean values of each group) were determined by an unpaired Students *t*-test (*z*-test), ANOVA, or a modified Fisher's exact test. Nonnormally distributed data were analyzed by the Mann-Whitney *U* test. 

## 3. Results

### 3.1. Histological Characteristics of Pregnant and Lactating Mammary Gland

**Figure 1:**
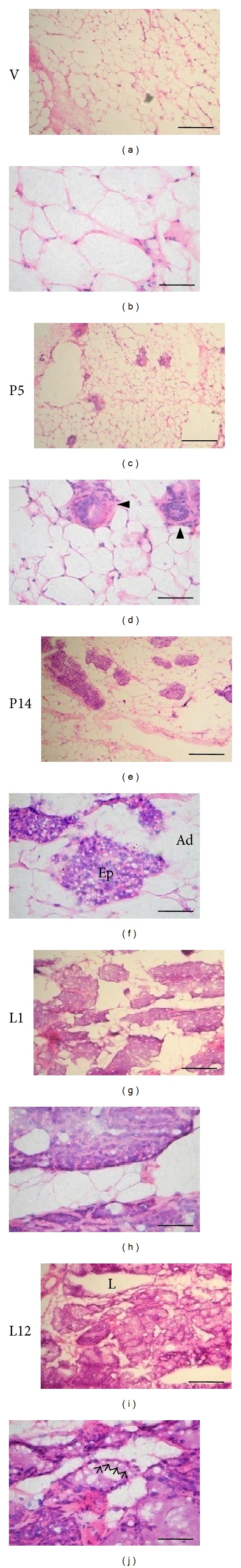
Histological features of the mammary gland from rats during pregnancy and lactation. Mammary glands were isolated from Sprague Dawley rats in (a, b) a nonpregnant virgin (V) stage; (c, d) day 5 (P5) and (e, f) day 14 (P14) of pregnancy; and (g, h) day 1 (L1) and (i, j) day 12 (L12) of lactation, cryosectioned, and stained with hematoxylin and eosin. Scale bars in (a), (c), (e), (g), and (i) = 50 *μ*m, whereas those in (b), (d), (f), (h), and (j) = 10 *μ*m. Adipose compartment (Ad), lobuloalveolar units (▴), epithelial compartment (Ep), luminal structures (L), and milk globules (↑).

The rat mammary gland undergoes a series of dramatic phenotypic changes during pregnancy and lactation. In order to determine the integrity of the dissected inguinal mammary glands, a gross histological evaluation of the characteristic cytomorphological features were determined through hematoxylin-eosin staining (Figure 1). Four time points (pregnancy days 5 and 14; lactation days 1 and 12) were selected to represent distinct periods in mammary gland development. Histologically, the mammary proliferative stage is represented by P5, the secretory differentiation stage by P14, early lactation by L1, and full lactation by L12.

As reported elsewhere [13], initial changes observed during pregnancy include an increase in ductal branching and the formation of alveolar buds (Figures 1(c) and 1(d)). The latter half of pregnancy is characterized by the expansion of alveolar buds to form clusters of lobuloalveolar units followed by the differentiation of these structures into presecretory structures. By day 14 of pregnancy, there is a readily apparent increase in the size of the epithelial compartment (Ep) (Figures 1(e) and 1(f)), and expansion of the epithelium (whereas the adipose compartment decreases) continues until the epithelial compartment predominates by onset of lactation (Figures 1(g) and 1(h)). By day 12 of lactation in the rat, the mammary gland is producing copious amounts of milk [7]. As expected, examination of the histology of the mammary gland at this stage reveals prominent luminal structures (L) and ducts and few adipocytes visible at this time (Figures 1(i) and 1(j)).

### 3.2. Global Analysis: Overview of Gene Expression

In this study, we analyzed expression profiles of 22,000 transcripts using murine microarrays and RNA samples of MG from virgin, pregnant, and lactating rats by cross-species hybridization. We first identified the total number of genes differentially expressed throughout distinct time points in mammary gland development such as P5, P14, L1, and L12.

A total of 807 oligonucleotide probe sets representing 521 annotated genes showed differential expression in at least one of four physiological time points evaluated, taking into consideration a mean *z*-score cutoff value of 1.50 standard deviations using GenArise. 

**Figure 2:**
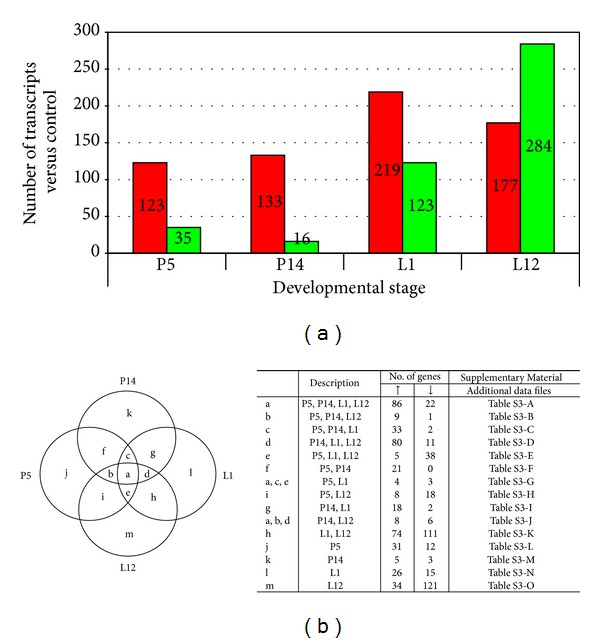
Overview of gene expression during mammary development through early pregnancy to mid-lactation. (a) Number of altered transcripts of each developmental stage versus a virgin stage (fold change (*z*-score) >1.5). Low expression is represented by green and high expression is represented by red. (b) Venn diagram and representative table illustrating the number of significant altered gene transcripts and overlaps among different reproductive stages.

During early pregnancy (day 5), 158 transcripts were differentially expressed. Most of these transcripts (123, 77.8%) were upregulated, suggesting a feasible tendency in the direction of gain of function versus the virgin stage (V). Likewise, in mid-pregnancy (day 14), as opposed to the virgin stage, the number of transcripts with an altered expression maintained a similar value (133 transcripts; 89.26% upregulated). During early and mid-lactation (days 1 and 12), 342 and 461 transcripts were differentially expressed, corresponding to a percentage of 64.0 and 38.4 overexpressed, respectively (Figure 2(a)). 

To further illustrate the differences and commonalities among the four physiological time points, changes in gene expression were also interpreted with a Venn diagram. As shown in Figure 2(b), the descriptive table of the Venn diagram denotes the number of genes showing upregulation (↑) or downregulation (↓) uniquely at pregnancy (day 5 or 14) or lactation (day 1 or 12) and differential expression at a combination of stages. Venn diagram analysis indicated that 47.2% (381/807) of all the differentially expressed transcripts presented an average significant *z*-score fold change (*z* > ±1.5) exclusively during either or both time points of lactation. Interestingly, among the 381 altered gene transcripts during lactation, 64.8% (247/381) were found downregulated, implying as previously stated by Lemay et al. (2007) [2] that mammary epithelial cells become biofactories not by gain of function but by a broad suppression of function to effectively push all cell resources towards a very few important tasks. All the gene sets that shared spatial and temporal distributions (overlapping changes in expression) are listed in additional data files (Table S3).

### 3.3. Clustering Analysis for Gene Expression

**Figure 3:**
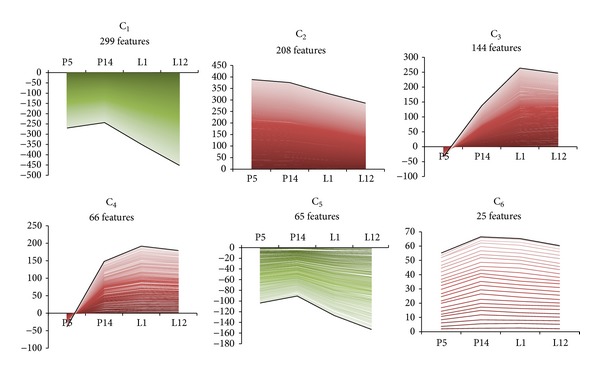
Unsupervised hierarchical clustering of the differentially expressed gene list. Gene expression profiles. Gene expression data from the 807 tags comprising the significant altered gene transcript list (*z*-score > ±1.50) were best represented by six clusters consisting of distinct up (red) and down (green) patterns of expression. Developmental stage time points and fold change are indicated on the *x*- and *y*-axes, respectively. The number of features (genetic elements) in each cluster is indicated. A black pseudoline representing the general (average) pattern of expression has been superimposed on each cluster.

To determine global alterations in gene expression across developmental stages of the mammary gland from early pregnancy to mid-lactation, we performed a complete-linkage hierarchical clustering with an Euclidean distance similarity metric on the expression profiles of the differentially expressed genes (annotated and EST) across all four time points. The expression profiles of the 807 genetic elements resulted in six predominant clusters on a dendogram (designated clusters 1–6 in Figure 3). All the gene cluster sets are enumerated in Table S4.

Cluster 1 (C1) represents 37.35% of the total 807 genetic elements. This major trend is a decline in gene expression during mid-pregnancy that remains low during lactation. Cluster 2 (C2) describes 25.77% of the total data population and is characterized by a linear decrement in gene expression towards mid-lactation. However, positive *z*-score values are retained with respect to the reference stage (virgin). The remainder of the clusters (C3–C6) appears to explain between 3.09 and 17.84% of the data variation. In C3 and C4, gene expression rises exponentially from early pregnancy, reaching a plateau during mid-lactation. However, the slope of the curve is even steeper in C3 in comparison to C4. In cluster 5 (C5), 65 elements matched the profile outline (inverted sigmoid form) of major trend C1 although the reduction tendency was less marked. In cluster 6 (C6), expression was roughly unchanged during pregnancy and lactation. Even so, the relative abundance of transcripts remained in a higher proportion than the reference virgin stage as described for C2.

This transcriptional profile, involved in the mammary development program identified in rat, could be conserved in others mammals like mouse. Consequently, in order to delineate potential groups of coregulated genes, final cluster membership was determined by a K-means analysis based on the preestimated number (six) of gene clusters.

**Figure 4:**
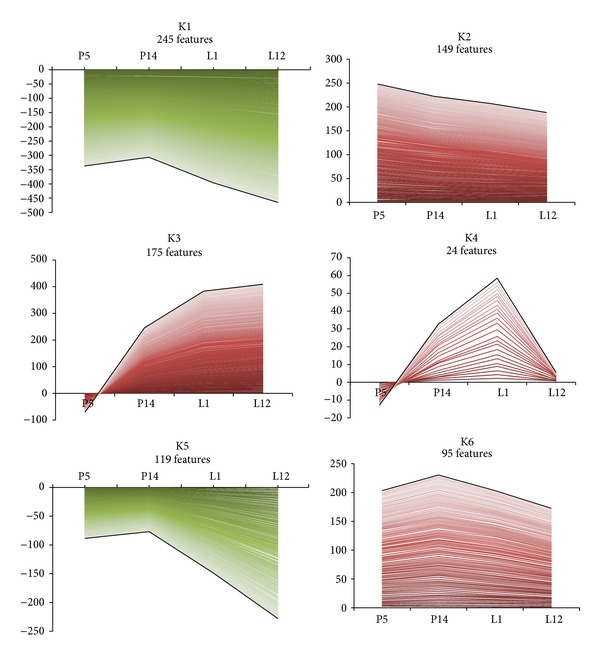
K-means clustering of the differentially expressed gene list with trajectory expression profile signatures. Gene expression data from the 807 tags comprising the significant altered gene transcript list (*z*-score > ±1.50) were best represented by six K-means clusters consisting of distinct up (red) and down (green) patterns of expression. Developmental stage time points and fold change are indicated on the *x*- and *y*-axes, respectively. The numbers of tags in each cluster are indicated. A black pseudoline representing the general (average) pattern of expression has been superimposed on each cluster.

K-means clustering revealed six distinct clusters (K1–6) that distinguished up from down, early from middle, and transient from sustained changes in expression (Figure 4; Table S5). Each of the six clusters was designated with its unique trajectory expression profile signature across stages (pregnancy days 5 and 14, lactation days 1 and 12) as presented in Figure 4. 

There were two major groups of 245 under- and 175 overexpressed tags during lactation only (Table S5, K1: 1,1,0,0 and K3: 1,1,2,2 according to the procedure of Rudolph et al., [10] to code the mean expression, see Section 2). Among the typical upregulated genes of lactation stage are the milk protein (casein alpha (*Csn1s1, Csn1s2a*), casein beta (*Csn2*), and whey acidic protein (*Wap*)) and biogenesis genes that mainly concern glucose and lipid metabolism (*Akr1c6, Aldob, Ugt2b1*, *Plb1, Apoe,* and *Sult2b1*) and transcriptional regulation (*Stat5a, Pou2af1*) [2]. Among those genes found significantly downregulated only during lactation, several play an important role in the regulation of apoptosis, mediation of metastatic behavior (epithelial-mesenchymal transition), or ubiquitin-mediated protein catabolism (lysosome degradation) in the mammary gland including *Igfbp5, Mmp2,* and *Ube2r2* [14, 15]. One hundred forty-nine genes were upregulated exclusively during early pregnancy (K2: 2,1,1,1) such as *Esr1, Esr2, Tshr,* and *Oxt.* These participate in the transduction of hormonal status [2, 16] involved in the modulation of important physiological processes such as carbohydrate metabolism (*Creb3l4, Hk1*, and *Coasy*) [17]; glutathione metabolism (*Ggt1, Mgst1,* and *Gstm6*) [18]; cell differentiation (*Foxa1, Mtap7, Gdf1, Twist2, Hey1, Dll4,* and *Pcaf*) [19–23], stromal-epithelial communication (cell-cell junctions) (*Cldn10, Mpp5,* and *Epb4*) [24], and cell adhesion (*Matn1, Krt71, Mpzl2,* and *Dscaml1*) [25]. 

The smallest group of 24 genes were significantly upregulated exclusively at the onset of lactation (K4: 1,1,2,1) such as *Lpo, Cd8a, *and *Irs1*, important for lactogenesis, particularly in milk production capabilities and related immunotropic constituents (antigen-specific CD8+ T cells) found in colostrum [26, 27]. One hundred nineteen genes were downregulated from early pregnancy (K5: 1,1,1,0). For example, *Acta1, Flnc, *and *Pax7*, which are either restricted to muscular tissues or involved in myogenic development and cellular differentiation [28, 29], are included in this group. According to the trajectory profile signature, 95 additional genes were found upregulated at all stages evaluated (K6: 2,2,2,2). Interestingly, most of the overexpressed genes in this group include general transcription and translation (including spliceosome and protein folding) machinery factors (*Eif4a2, Eif2ak1, Etf1, Taf1, Ercc2, Sart1, Ppih, *and *Dbr1*) [30, 31] as well as structural (*Itga5, Actg1*, *Add2, Cldnd2, Rptn,* and *Triobp*) [32] and basal metabolic genes (*Pank4, Agpat5, Cyp24a1,* and *Phyh*) [33]. 

### 3.4. Determination of Orthologous Genes

Once the gene clusters were properly defined, identification of orthologous gene transcripts among the time course differentially expressed gene list subsets was critical for reliable comparison of gene function and subsequent determination of probably conserved transcriptional modules implicated in biological processes during the development of mammary tissue. According to genome comparative RoundUp orthologous database of a total of 448 transcripts upregulated and 371 downregulated, 213 (upregulated) and 183 (downregulated) genes were identified as orthologous to rat and/or human. The remainder of the genes was discarded or removed from subsequent analysis due to lack of similarity, insufficient information, or unknown identifiers. A complete list of orthologous genes from each dataset was compiled (Table S6).

Among the upregulated orthologous genes to rat and/or human are those encoding to milk proteins, carbohydrate and lipid metabolism, transcriptional factors [17, 34], transduction of hormones [16], glutathione metabolism [18], and cell differentiation [19, 20]. Others are associated with stromal-epithelial communication [24], cell adhesion [25], lactogenesis [26], and general transcription and translation machinery factors [30] as well as structural [32] and basal metabolic genes [33–35]. 

Among those genes found significantly downregulated, several play an important role in the regulation of apoptosis, mediation of metastatic behavior (epithelial-mesenchymal transition), or ubiquitin-mediated protein catabolism (lysosome degradation) [14, 15]. Also, genes restricted to muscular tissues or involved in myogenic development and cellular differentiation [28, 36] are downregulated.

### 3.5. Confirmation Studies

**Figure 5:**
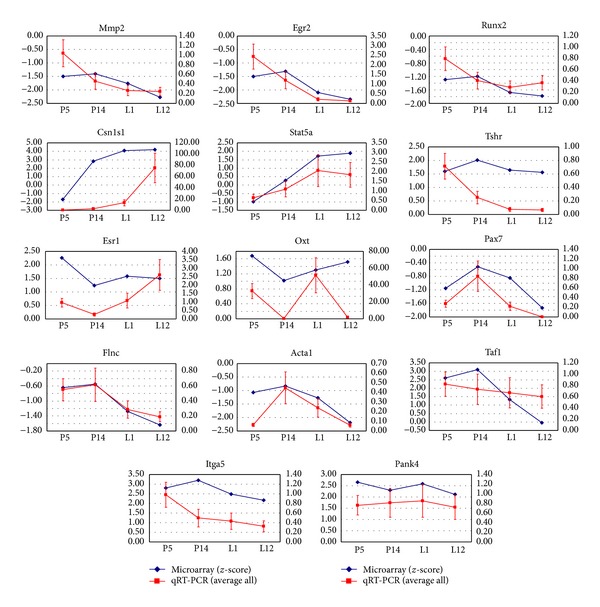
Validation of differentially expressed genes identified by microarray using qRT-PCR. Quantitative real-time-PCR was performed as in *Experimental Procedures*, and the calculated mean log ratio for each gene in their corresponding developmental stage was compared with the mean *z*-score from the microarray analysis during that same period. A positive value indicates a greater mRNA abundance in a developmental stage than in the control group, whereas a negative value indicates a lower mRNA abundance in a developmental stage than in the control group. Lines represent data obtained by qRT-PCR (red, *right *axis) and microarray analysis (blue, *left *axis), respectively, whereas the *x*-axis represents the physiological time point. The average fold change relative to time-matched virgin controls for three animals per group is shown. Genes are indicated by their official gene symbols. Data are presented as the mean of three independent experiments, each performed in triplicate. Error bars represent the SD for the average fold change. The correlation information in each analysis is indicated by Spearman rho value (*r*_*s*_). All 18 pairs of genes had an absolute correlation value of *r*_*s*_ = 0.49 with a *P* = 7.59*E* − 06. ND: not detected.

Taking into consideration their temporal expression profile signature and the fact that they represent different K-means cluster, 14 genes were selected for real-time PCR analysis (Table S2). Results show that the expression trends were consistent with the results from the microarray analysis. Correlation analysis showed good agreement between real-time RT-PCR and microarray analysis. Microarray results for all 14 genes tested were confirmed by real-time RT-PCR with regard to direction and significance of change (Figure 5). 

## 4. Discussion

Structural and functional homologies of specific genes are important. Conservation of functional blocks of genes is likely to be more important in a cross-species comparison. We found distinct blocks of significantly differentially expressed genes within different cytogenetic regions of the rat with homologous chromosomal segments in human and mouse. However, human, mouse, and rat have different chromosomal arrangements. Genes in these blocks appear in contiguous cytogenetic regions, irrespective of species and chromosomal location. This finding is not surprising considering the close evolutionary distance between the species where 278 orthologous segments are reported to be shared between human and rat, and 280 segments are reported to be shared between human and mouse [4]. It is proposed that these gene blocks may be significant for mammary gland development and maintenance and progression of lactation across human, rat, and mouse. For example, genes in the blocks may be coordinately expressed to share transcription programs as stated in previous studies [37]. The argument may be made against the feasibility of using rodent data to draw inferences to human mammary gland gene expression. However, our objective in this study was to utilize the best available sources of information such as rat gene expression data during mammary development and mapping data to develop hypotheses on putative functional gene blocks conserved across species, underlying similarities despite reported differences in the architecture and hormonal control of mammary glands between rats and other rodents and between rats and humans [38, 39].

**Figure 6:**
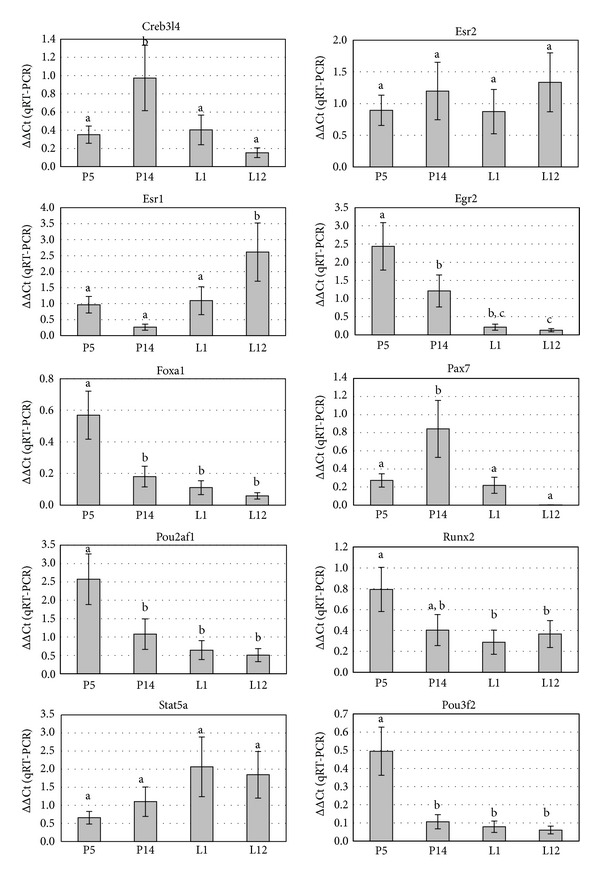
Gene expression profiles of the putative transcriptional factors identified by microarray using qRT-PCR. Quantitative real time-PCR was performed as in *Experimental Procedures*, and the calculated mean log ratio for each gene in their corresponding developmental stage was determined. A positive value indicates a greater mRNA abundance in a developmental stage than in the control group, whereas a negative value indicates a lower mRNA abundance in a developmental stage than in the control group. Bars represent data obtained by qRT-PCR (*y*-axis), whereas the *x*-axis represents the physiological time point. The average fold change relative to time-matched virgin controls for three animals per group is shown. Genes are indicated by their official gene symbols. Data are presented as the mean of three independent experiments, each performed in triplicate. Bars represent the average (±SD) fold change. Different letters indicate statistically significant differences between developmental stages (*P* < 0.05). Note that the scales of the *y*-axis vary among genes. ND: not detected.

In an effort to further characterize potential highly coregulated gene blocks, we combined transcription-factor binding-site prediction [40] along the promoters of each gene member with the detection of expression profiles of annotated altered transcripts categorized as nucleic acid binding protein. Several families of transcription factors were identified (Table S7). For the most part, zinc finger domain/motif proteins were the most widely represented. The presence of *cis-*elements found with CORE_TF (http://www.LGTC.nl/CORE_TF) in the promoters of the genes *Slc44a4, Ppt2,* and *B3galt4* that compromises the conserved block D15 (Table S8) along with the cotranscription of mRNAs that encoded for *trans-*regulator elements suggests that they are most likely modulated by transcription factors *Runx2, Creb3l4, Pou3f2,* and *Pou2af1*. Correspondingly, the gene members of block U1 may possibly be coregulated by transcription factors *Stat5a, Foxa1, Creb3l4,* and *Pou2af1.* In the same manner, other gene blocks (U3, U8, U9, D1, D9, and D14, Table S8) were found most likely co-regulated by a minor number of transcription factors (*Foxa1, Creb3l4, Pou2af1,* or *Egr2*). Hence, identification of conserved overrepresented upstream motifs unravels putative regulatory elements for transcription (Figure 6) in at least half of the gene block members reported in this study. Consequently, these results strongly substantiate the maintenance of comparable transcriptional regulation programs among the predicted coexpressed modules.

Because cotranscription of genes in conserved blocks may allow concerted expression of gene products involved in the same response or pathway [41], integration of this type of analysis enables the discovery of putative evolutionary conserved regulatory networks among mammals. Thus, the co-regulated clusters we proposed may indeed be conserved transcriptional modules through evolution, at least between rodents and primates. 

Heterologous hybridization experiments on any microarray are of limited use for genes that have undergone rapid evolutionary change in coding regions, large rearrangements, and duplication [42]. Long oligonucleotide-based microarray platform may be more suitable for cross-species gene expression studies than a short oligonucleotide-based system [43]. This comparative approach is based on the assumption that similar gene sequences in closely related species allow a reasonably reliable detection of many orthologous genes. For instance, according to several independent and unrelated studies carried out on comparable 50 to 60-mer oligonucleotide microarrays, cross-hybridization was observed only with genes with 50%–75% overall sequence identity, respectively [44, 45]. Considering that orthologous genes between human and mouse and between human and rat both have a mean of ~85% sequence identity [46], validity of the results obtained in this study—despite the problems encountered by CSH—in comparison to SSH seems upholding. In fact, the nucleotide sequence alignment confirmed an >75.3% homology at least for the transcript members of the distinct gene blocks described, depending on the sequence evaluated among primates and rodents reinforcing the notion of attaining valid biological results. In addition, similar expression trends for distinct probe sets for one corresponding gene (data not shown) seem to largely substantiate the certainty and reproducibility of hybridization results obtained in this study.

Because of the challenges inherent to CSH data, their confirmation by other techniques is essential [43]. In addition to qRT-PCR, orthologous gene expression profiles with syntenic regions of rat, mouse, and human chromosomes reinforce another confirmation method that potentially substantiates the CSH results obtained in this study. Nonetheless, further validation of the results must be carried out by using CSH of human RNA to mouse oligonucleotide arrays. 

This study provides access to a prevalidated platform for analyzing transcriptional changes in rat mammary gland. This paper will hopefully spur an increase of mammary gland CSH transcriptome analysis, thus adding to our knowledge base of this interesting evolutionary feature among mammals. However, although we acknowledge the multitude of aspects that can be elucidated by traditional SSH transcriptome analysis, we believe the biggest potential of the presented microarray lies in the multispecies-type studies described. We demonstrated that data analysis strategies such as the combination of orthologous gene expression profiles and chromosome mapping in conjunction with directed promoter transcription-factor binding-site prediction presented here can add strength to conclusions and help identify systems and responses that are conserved across the mammal *taxa*. The possibility of studying the evolutionary depth of transcriptional regulation adds a new dimension to comparative transcriptomic, particularly identification of differentially co-regulated gene blocks mapped to highly conserved syntenic chromosomal regions, which is important in mammary gland development using CSH experiments among mammal species.

## Supplementary Material

This manuscript contains supplementary material to give a better overview of the results. In Table S1 we are describing the experimental design of heterologous microarrays hybridization; in Table S2 we showed the primers and probes for the qRT-PCR analysis for confirmation of microarray results. In S3 we are showing the list of up- and downregulated genes during pregnancy (P5, P14) and/or lactation (L1, L12) that shared spatial and temporal distributions. In S4 we described all the gene hierarchical cluster sets of altered gene transcript. In S5 is the list of altered gene transcript K-means cluster sets that correspond to potential groups of co-regulated genes. K-means clustering revealed six distinct clusters (K1-6) that distinguished up from down, early from middle, and transient from sustained changes in expression. The data from Table S6 shows the list of orthologous significant altered gene transcripts to rat and/or human in pregnancy (day 5 and 14) and lactancy (day 1 and 12). The Table S7 shows the list of families of transcription factors identified in different stage development of mammary gland. Finally in Table S8 we are describing the functional gene blocks detailed information.
